# Do you have enough space? Habitat selection of insectivorous cave-dwelling bats in fragmented landscapes of Eastern Amazon

**DOI:** 10.1371/journal.pone.0296137

**Published:** 2025-01-09

**Authors:** Valéria da C. Tavares, Mariane S. Ribeiro, Xavier Prous, Alice A. Notini, Nathalia Y. Kaku-Oliveira, Leandro M. D. Maciel, Sérgio Sales, Juliana M. Longo, Flávia M. Evangelista, Lucas Rabelo, Iuri V. Brandi, Santelmo S. Vasconcelos, Sonia S. Talamoni, Guilherme Oliveira, Leonardo C. Trevelin

**Affiliations:** 1 Instituto Tecnológico Vale (ITV), Belém, Pará, Brazil; 2 Pós-Graduação em Biologia (Zoologia), PPGCB, Depto. de Sistemática e Ecologia, Universidade Federal da Paraíba (UFPB), João Pessoa, Paraíba, Brazil; 3 Museu Paraense Emílio Goeldi, MPEG, Pós-Graduação em Biodiversidade e Evolução, Belém, Pará, Brazil; 4 Environmental Licensing and Speleology, Vale S.A., Nova Lima, Minas Gerais, Brazil; 5 Programa de Pós-Graduação em Biologia de Vertebrados, Pontifícia Universidade Católica de Minas Gerais, Belo Horizonte, Minas Gerais, Brazil; 6 Ativo Ambiental, Belo Horizonte, Minas Gerais, Brazil; Southeastern Louisiana University, UNITED STATES OF AMERICA

## Abstract

Individual movements of bats are triggered by their life requirements, limited by their recognition of the environment and risks of moving, and mediated by habitat selection. Mining adds fragmentation and heterogeneity to landscapes, with poorly understood consequences to the life activities of the bats. Cave dwelling bats spend most of their life cycles within caves, and as they constantly forage in external landscapes, their contribution in the input of organic matter to the caves is of paramount importance to the subterranean biodiversity. We investigated habitat selection by cave bats in a mosaic of Amazonian forests, iron-rich savannas and iron-caves, studying the movements of the aerial insect-catcher *Furipterus horrens* (Furipteridae) and of the foliage gleaning bat *Lonchorhina aurita* (Phyllostomidae), both cave-dependent in the area. We used radio telemetry to assess their use of habitats, under the prediction that these species prefer preserved habitats for their foraging bouts, avoiding human-modified habitats. We also anticipated larger areas and commuting distances for both species when foraging in disturbed landscapes. Thirty-one bats were monitored in conserved habitats, mining sites and pastures resulting in an average range of 415.1 ± 837.4 ha and average commuting distances of 2163 ± 2453 m (*F*. *horrens*) and 681.1 ± 1101 ha and 2781.5 ± 2333 m (*L*. *aurita*). Our results suggest that *F*. *horrens* are open space foragers, frequently recorded in cangas, and *L*. *aurita* are forest foragers that use all habitats proportionally to their availability in the landscape. We detected landscape-related effects mediated by seasonal variation in the maximum commuting distance performed by both species. These are the first radiotelemetry data available for *F*. *horrens* and *L*. *aurita*, delivering original baselines to understand their movement behaviors. This information sheds light into constraints related to the optimal and adjusted biological cycles of these two species and their range shifts under scenarios of disturbance and may subsidize programs for their conservation.

## Introduction

The behavior of organisms in the wild is expressed as a compromise among sets of possible responses, enabling them to cope with adverse situations, such as those arising from anthropogenic activities [1]. Movement behaviors result from the conciliation between the need of exploration of the environment to meet life requirements with preventive actions related to potential threats associated to moving [2, 3]. Those trade-offs are mediated by habitat selection that enhance the chances of individuals and species to optimize their performance and fitness regarding their use of space [4]. Understanding habitat selection can shed light on behavioral mechanisms that allow species to exploit heterogeneous environments [5] and provide insights into the influences of habitat quality on individuals and populations [1].

Cave-dwelling bats spend roughly half their life cycles roosting underground for resting, mating, and raising offspring [6, 7], and the other half searching for and gathering resources during night foraging bouts in the external landscapes [8]. The energetic requirements of bats for their daily activities and the heterogeneity of the landscapes available for their foraging movements are among the main limiting factors shaping these nightly outside hunts driven by habitat selection [9].

Although caves have usually been the focus of conservation efforts for the cave-related biota [10], the surrounding landscapes can encompass most of the home range of a bat. These interplays between the underground and the surrounding landscapes shapes overall biodiversity patterns in subterranean environments worldwide [7] and are pivotal to maintain the diversity and abundance of cave bats over time [11]. The same holds true for subterranean invertebrate assemblages, which are largely sustained by the guano deposition made by bats [8, 12].

The conservation of subterranean ecosystems is a contemporaneous challenge. Given their unique biodiversity, often concentrated in areas rich in mineral resources [13], cave-associated ecosystems are frequently vulnerable to intense and multi-scale landscape modification. The consequences to the underground biodiversity are poorly understood, and this demands concentrated efforts including the unveiling of biological patterns of these systems and to provide indicators that convey agile and reliable insights to monitoring programs focused on caves and their associated landscapes [14–16]. Understanding habitat selection patterns of cave-dwelling bats can provide a baseline to bat monitoring and to obtain timely evidence of changes in the bat movement behavior that may be associated to environmental changes [1] and subsidize conservation and management plans to cave bats and to the associated cave biodiversity [8].

Understanding how bat movements are affected by habitat loss and fragmentation [e.g.^17^, ^18^] remains challenging. Data on the use of space, habitat preferences and selection by bats have seldom been assessed in the Neotropics e.g. [19–23]. Information is even rarer for the bats with Amazonian distribution and virtually non-existent for bats in tropical cave systems. The scarce evidence available suggests that seasonality modulates Amazonian bats’ responses to landscape structure, signaling the role of spatiotemporal variation of food resources as a template for the foraging and movement behavior of bats [24, 25]. These patterns are complex, and they are likely species and ensemble-specific (e.g. foraging guilds) responses [26], and better understanding them is critical for karstic and pseudokarstic landscapes demanding management actions for habitat protection, such as the unique iron cave complexes from Eastern Amazonia.

One of the world´s most extensive iron cave systems occurs in the Eastern Amazonia region of Carajás. The heterogeneous landscapes of Carajás concentrate over 1500 iron caves in a mosaic of well-preserved Amazonian forests and iron-rich cangas with areas disturbed by iron-mining and peri-rural activities, all primarily enclosed within the Carajás National Forest, and harboring a rich bat fauna [27]. Some of these species are regionally cave-dependent, such as the phylogenetically distant and morpho-ecologically highly distinctive insectivorous species, the thumbless bat, *Furipterus horrens* Bonaparte, 1837 (Chiroptera: Furipteridae), and the common sword-nosed bat, *Lonchorrina aurita* (Chiroptera: Phylostomidae).

Thumbless bats (*F*. *horrens*) are tiny (3–4 g) and have a delicate appearance, broad wings, a long tail extending for most of their large uropatagium, and small thumbs enclosed by their wing membranes. Populations of *F*. *horrens* are generally associated with humid forested areas and limestone rocky shelters, forming groups that range from small to large colonies. These bats are distributed from Venezuela to the transition between the semideciduous forests and the Caatinga of Southern and Northeastern Brazil [28, 29]. They have been found inhabiting rock outcrops in the Xingu riverbed [30] and forming small groups, often including juveniles, in fallen trees in forests of French Guyana [31]. In Carajás, *F*. *horrens* relies heavily on the iron caves for roosting, forming large aggregations [30]. The biology of *F*. *horrens*, considered one of the rarest Neotropical bat species, is scarcely known [29]. Most of its known South American range and records are within Brazil, where it has been listed as “Vulnerable” based on rough estimates of the percentage of mature individuals per population and based on its association with karstic landscapes under the influence of mining activity, cattle ranching, habitat fragmentation, and urban expansion [32]. *Lonchorhina aurita* Tomes 1863 (Phyllostomidae) is a medium-sized foliage-gleaning insectivorous bat (12–16 g), categorized as “Near threatened” in Brazil [32], that possesses a singular echolocation system, which is considered an adaptation to aerial hawking [33]. This species has been found roosting in tree hollows [34], but like *F*. *horrens*, it extensively occupies iron caves in Carajás [27]. For both *F*. *horrens* and *L*. *aurita*, virtually nothing is known about their use of space and habitat selection.

Our two-fold goals in this study are to (1) describe movement data of the bat species *F*. *horrens* and *L*. *aurita*, generating baseline information on their use of space and habitat selection patterns, and (2) estimate responses of these two bat species to anthropogenic disturbance at landscape levels. We hypothesized that these two species of insect-feeding cave bats would rely mainly on well-preserved forest and canga habitats for their foraging activities and used radiotelemetry to assess their habitat selection modes. We predicted the avoidance of anthropogenic, modified habitats by *F*. *horrens* and *L*. *aurita*, such as mining and pastures while foraging [10], and that the range areas and commuting distances would be larger in disturbed landscapes for both species [20].

## Methods

### Study site

**Fig 1:**
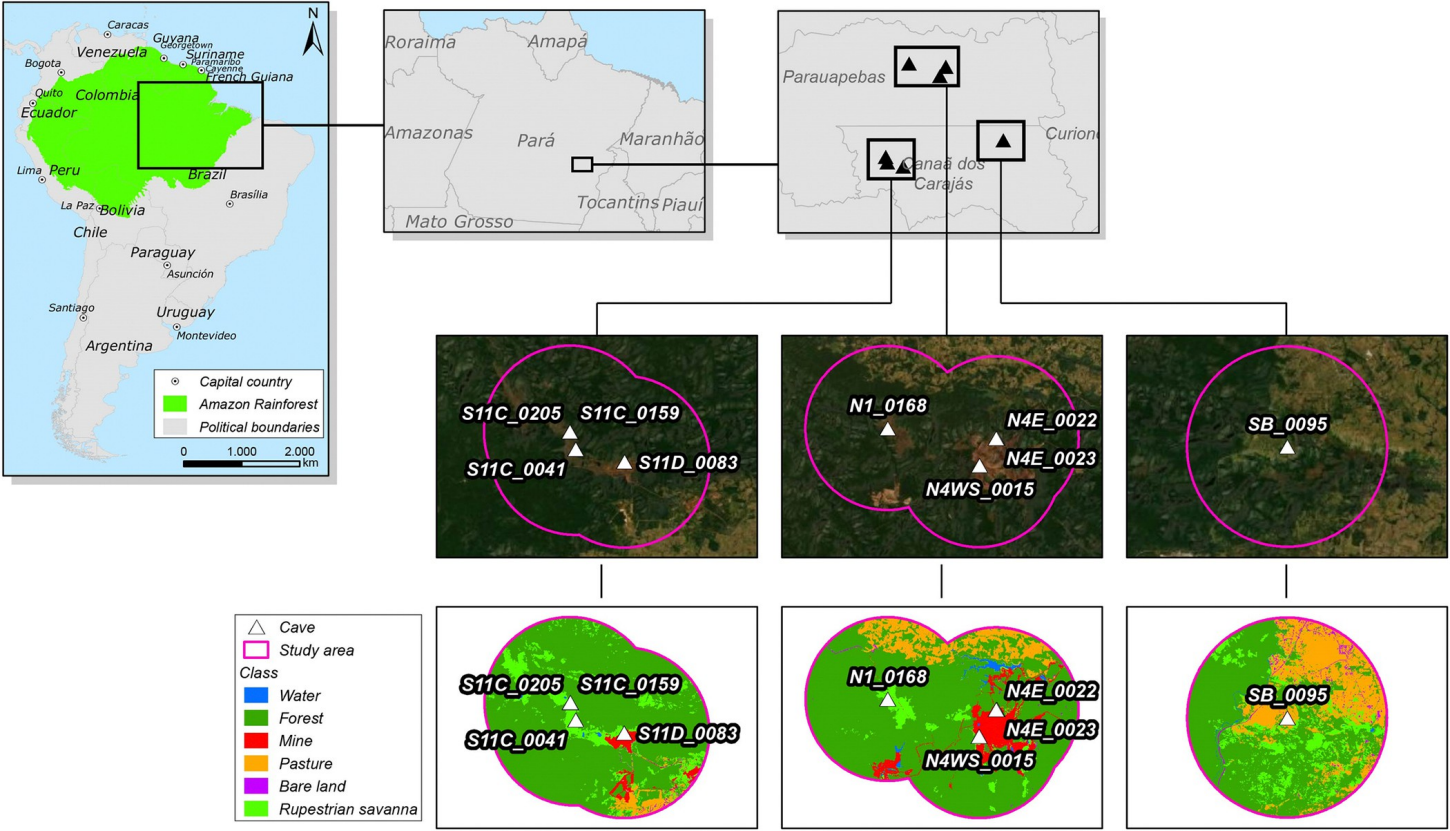
Location of the study area, the Floresta Nacional de Carajás, and study sites containing the caves sampled and the habitats considered in the study, present in the surrounding landscapes. Contains Copernicus Sentinel data [2021] processed by Sentinel Hub.com.

We studied the movements of *F*. *horrens* and *L*. *aurita* in the National Forest of Carajás (FLONA Carajás), and in the Campos Ferruginosos National Park, two contiguous Brazilian protected areas located in the Carajás mountain range of southeastern Amazonia, state of Pará, Brazil. Carajás encompasses complex subterranean ecosystems with over 1500 iron caves already prospected [35] and, given to its vast and unique iron-rich terrains, it has also been considered one of the world’s largest deposits of high-grade iron ore [36]. The region is composed of medium altitude plateaus (between 500 and 700 m) scattered along lowlands and forming a mosaic of typical Amazonian rainforests and rocky outcrops with savannah-like vegetation. These savannah-like ecosystems are however uniquely adapted to iron-rich soils, dissimilar to other Amazonian savannas, and called “cangas” [35, 37, 38]. The Carajás region currently combines pristine and recovering protected areas and areas for ore exploitation (iron, copper, manganese) and other unprotected areas under anthropogenic use such as pastures, abandoned crops and peri-urban zones [39]–Fig 1). The regional climate is defined as Aw following Köppen classification, marked by high annual rainfall with a well-defined dry season [40].

### Experimental design and data collection

#### Landscape data

The composition of the landscapes surrounding each of the study areas was initially accessed within a circular 10 km-radius buffer centered around the main cave entrance, using photo-interpreted data from Sentinel 2 images with 10 meters of spatial resolution. The final buffer size was based on the farthest bat location detected by our equipment and according to our sampling protocol, and it was an empirical estimate of the maximum landscape area that we effectively sampled [20]. We discriminated six landcover classes as landscape habitat categories and quantified their availability within each buffer: four natural habitats (forest, canga, water bodies and exposed bare land) and two anthropogenic land uses (mining and pastures). Our experimental design was then composed of three categories of landscapes (treatments) based on the presence and proximity of the cave sampled to anthropogenic habitats: landscapes exclusively dominated by preserved natural habitats (forests and savannah-like “canga”, less the ~5% of anthropogenic habitats), landscapes containing mining land use (> 5% mine habitat close to the cave) and landscapes dominated by pastures (> 20% of pastures habitat) (Fig 1). All imagery analyses were conducted using QGIS 3.14. We based the final selection of caves for our study combining the presence of our target species (*F*. *horrens* and *L*. *aurita*) and the representativeness of the surrounding landscapes within our experimental design.

#### Capturing and marking bats

**Fig 2:**
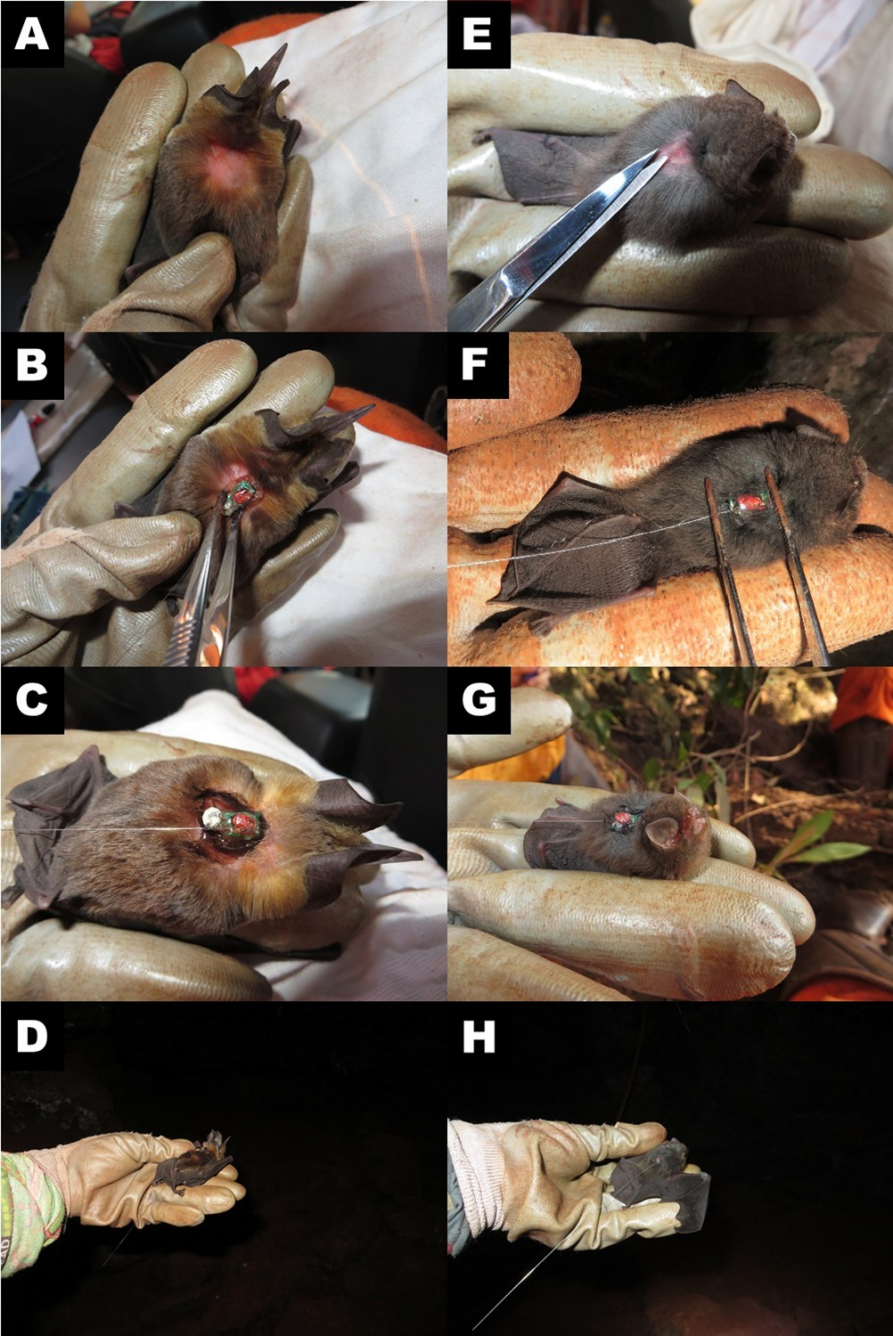
Attachment of radio transmitters, after the clipping of the fur, and release of the bats: A) Hair clipping in the back of a *L*. *aurita* individual; B) gluing of the transmitter to the back of a *L*. *aurita* individual; C) Transmitter glued on and D) hand release. E) Hair clipping in the back of a *F*. *horrens* individual; B) gluing of the transmitter to the back of a *F*. *horrens* individual; C) Transmitter glued on, and D) hand release. All images were taken by the authors.

Bats were captured during the daytime while roosting inside caves using entomological hand nets [41], as we wanted to make sure that we would be monitoring the movements of cave residents. The individuals monitored for each species were treated as replicates of their populations, and each population was sampled twice to encompass the wet and the dry seasons. We tagged selected individuals of *F*. *horrens* and *L*. *aurita* with radio transmitters with unique signaling frequencies (LB-2x –Holohil Inc., Canada). For every individual captured we first took measurements and weight, and additional information such as sex and life stage (juvenile or adult). For the females we also annotated reproductive condition (pregnant and/or lactant) [42]. We carefully trimmed patches of upper middorsal fur, close to the scapula horizontal plane (in relation to a longitudinal body axis) to attach the radio transmitters with the help of surgical adhesive (Fig 2). The weight of each transmitter was 0.31 to 0.41 g, corresponding roughly to 3% of *L*. *aurita*’s and 8% of *F*. *horrens* total body weights. The latter value is above an ideal threshold of 5% [43] but remains below the 10% threshold considered adequate for short-term studies and for the small size of the species [44, 45]. We released radio-tagged bats inside the caves in which they were originally captured and began to record radiotelemetry data departing from the next night to avoid bias resulting from the capture and handling of individuals. Transmitters fixed with surgical adhesive typically remain attached for fewer than 10 days [44], close to the minimal battery duration expected for the models we used. Thus, in our study, we retrieved some transmitters after detachment using positioning techniques (see below). However, our experimental design precluded us from retrieving most transmitters due to battery failure.

We selected only healthy adult and non-reproductive females to receive radio transmitters, as indicated by our physical examination of the bats. We complied with the guidelines from the American Society of Mammalogists [46] for the use of wildlife in research, and this study was authorized by the Instituto Brasileiro do Meio Ambiente e dos Recursos Naturais Renováveis (IBAMA) (ABIO N° 1000/2018).

#### Radiotracking

We conducted six sampling sessions, three in the dry season (August and September 2018, July 2019) and three in the wet season (November 2018, February and November 2019). We set a sampling protocol for each marked individual, in which we divided every sampling night into three to four hours intervals, starting at 6 PM and ending at 6 AM of the next day. Our aims were to sample all intervals for all individuals in a way to allow for inferences about habitat use in situations where the number of location fixes was different among individuals (replicates) [20, 47]. Independent teams conducted signal monitoring by performing active radio signal searches and bi-angulation techniques to obtain locations [48] using a radio-receiver model TRX-1000 (Wildlife Materials Inc.) coupled to three-element Yagi antennas. We mapped successive locations and roost fixes to obtain flight routes and range areas. Based on our protocol and raw data exploration, we considered one individual as successfully monitored only when it was detected and monitored in all night intervals and sampled for more than one day and nine locations. We initially marked and tried to monitor a total of 46 individuals, and only successfully monitored 30 due to loss of signal. A total of 16 *F*. *horrens* and 14 *L*. *aurita* were successfully monitored, with similar numbers of sampled individuals between seasons (see Table 1).

**Table 1:** Summary of results obtained per species. Species/Individuals, Sex, Forearm (length in mm), Cave, Treatment, Date, Season, Days (sampled) and N (Number of locations estimated). MCP (ha)–Minimal Convex Polygon estimate. KF 95% (ha)–Fixed Kernel estimates of range use with 95% (ha) of samples; KF 50% (ha)–Fixed Kernel estimates of range use with 50% (ha) of samples; MC 95% (m)–Maximum linear distance travelled from cave entrance in KF 95%; MC 50% (m)–Maximum linear distance travelled from the cave entrance to core foraging areas in KF 50%. At the bottom of each species, we present the average and standard deviation of estimates for each parameter related to the use of space.

Species/ Ind.	Sex	Forearm	Cave	Treatment	Date	Season	Days	N	MCP (ha)	KF 95% (ha)	KF 50% (ha)	MC 95%	MC 50%
*Furipterus horrens*		* *	** **	** **	** **	** **	** **	** **	** **	** **	** **	** **	** **
1	male	34.8	N4WS_0015	Mining	ago/18	Dry	2	34	14.9	40.0	8.4	983	845.5
2	male	33.9	N4WS_0015	Mining	ago/18	Dry	2	26	15.8	47.9	12.5	936	763.8
3	male	35.5	N4WS_0015	Mining	ago/18	Dry	3	21	25.7	96.0	25.5	965	772
4	male	36.8	N4WS_0015	Mining	ago/18	Dry	2	30	25.6	86.4	17.3	1068	394
5	male	34.5	SB_0095	Pasture	set/18	Dry	3	35	4.3	404.2	64.8	1691.6	510.6
6	female	36.1	SB_0095	Pasture	set/18	Dry	3	23	1.1	3.0	0.5	366	132.6
7	male	34.4	SB_0095	Pasture	set/18	Dry	2	22	53.8	192.4	53.7	1239.5	846.2
8	male	35	S11C_0041	Preserved	out/18	Dry	9	62	309.0	670.4	155.8	3283.9	2630.3
9	male	35.2	S11C_0041	Preserved	out/18	Dry	5	60	38.0	72.3	18.3	1201.6	878.5
10	male	-	SB_0095	Pasture	nov/18	Wet	5	43	37.8	60.8	8.7	2002.4	275.2
11	female	-	SB_0095	Pasture	nov/18	Wet	3	63	39.5	74.1	9.7	1281.8	327
12	male	-	SB_0095	Pasture	nov/18	Wet	5	56	263.4	728.7	113.1	4352.2	763.5
13	male	33.8	N4WS_0015	Mining	nov/18	Wet	2	57	1921.9	3545.2	500.9	10852.3	2094.5
14	male	34.3	N4WS_0015	Mining	dez/18	Wet	4	29	126.8	399.4	97.2	2515	1183.8
15	male	-	S11C_0205	Preserved	dez/19	Wet	5	112	54.3	44.5	7.0	774.4	410.8
16	male	-	S11C_0205	Preserved	dez/19	Wet	3	11	35.9	175.7	52.0	1107	812
Mean ± SD		34.9±0.9							185.5 ± 456.73	415.1 ± 837.4	71.6 ± 119.1	2163.7 ± 2453.1	852.5 ± 636.8
*Lonchorhina aurita*		* *											
1	male	46.9	N4WS_0015	Mining	ago/18	Dry	4	10	19.7	93.5	22.1	1345.4	1169.2
2	male	48.5	N4WS_0015	Mining	ago/18	Dry	6	48	111.1	163.1	21.5	1982	483.5
3	male	50	N4WS_0015	Mining	ago/18	Dry	4	19	80.2	196.9	53.6	1568	1280
4	male	47.7	N4WS_0015	Mining	ago/18	Dry	4	43	123.0	210.0	42.8	1883.6	1530.5
5	female	50	N4WS_0015	Mining	ago/18	Dry	4	14	40.3	177.6	50.4	1651.3	1384.6
6	male	49.8	SB_0095	Pasture	set/18	Dry	2	35	54.7	105.1	21.7	1463.5	872.3
7	female	48.6	SB_0095	Pasture	set/18	Dry	6	44	1259.5	2579.0	483.9	5148.6	3826.5
8	female	48.8	SB_0095	Pasture	set/18	Dry	3	23	39.8	122.2	28.2	2352	1713.9
9	male	-	N4WS_0015	Mining	fev/19	Wet	5	12	160.2	566.3	125.6	2168.2	1543.7
10	male	-	N4WS_0015	Mining	fev/19	Wet	3	20	47.3	155.0	42.7	1371	961.4
11	male	-	N4WS_0015	Mining	fev/19	Wet	5	41	450.9	741.1	133.2	5069.7	2293.4
12	male	-	SB_0095	Pasture	nov/19	Wet	4	32	4291.8	3947.5	514.5	9950.3	4663.7
13	male	-	S11C_0041	Preserved	nov/19	Wet	3	27	28.2	65.1	12.2	1268.8	422.5
14	male	-	S11C_0041	Preserved	nov/19	Wet	5	9	101.8	412.4	109.2	1718	1020.9
Mean ± SD		48.8±1.1							486.3 ± 1101.2	681.1 ± 1101.4	118.7 ± 160.0	2781.5 ± 2332.9	1654.7 ± 1166.3

### Data analysis

#### Range area estimates

We used Best Bi-angulation Estimators to determine locations, after correcting for magnetic declination in all compass bearings, using LOAS™ (Ecological Software Solutions, Inc.). Location datasets determined for each individual were input into ESRI ArcGis Desktop 10.2 software and range areas were subsequently estimated using the HRT 2 extension, Home Range Tools [49].

We generated estimates of range areas for each individual following Kernohan *et al*. [50], as the ‘‘extent of area with a defined probability of occurrence of an animal during a specified period”. We used fixed normal kernel density estimations (KF) with 95% (KF 95%) and 50% (KF 50%) kernel isopleths to delineate the area ranges [51] implementing least-squares cross-validation (LSCV) to estimate the smoothing parameter (H) [52]. We also generated range area estimates using Minimum Convex Polygon (MCPs) estimates, uniting the most external locations. These range estimates were used in our hypotheses testing.

#### Habitat use/selection

To evaluate habitat selection patterns, we estimated individual-based habitat uses by contrasting the proportional use of habitats by each individual with the estimates of habitat availability in the landscape. Since “habitat” is scale-dependent we organized our data following Johnson’s [53] hierarchical orders of habitat selection. In our framework, the second-order selection represents the proportional use of a determined habitat considering 95% kernel density estimations (KF 95%), and the third-order selection is the proportional of a determined habitat considering KF 50% kernel density estimations (KF), and both are nested within the MCPs estimated for each separated individual.

We used the non-parametric analytics as proposed by Fattorini et al. [54] based on the combination of sign tests, in which every habitat is individually tested. Following these analyses, overall statistic values for the simultaneous assessment of habitat selection in all habitat types can be obtained by combining P values from each test through permutation.

We considered all six habitat types: Forest, canga, mining land, pasture, exposed (bare) land and water bodies, and assessed whether each habitat was proportionally used according to its availability or whether it was over or under-utilized by each bat individually. All analytical procedures were conducted within the R environment [55], using package "phuassess" for R 3.3.1 [56].

#### Modelling use of space

To evaluate the effects of landscape composition in the movement behavior of the bats, we modelled their use of space using four parameters: range area (KF 95% area in hectares), core-foraging area (KF 50% area in hectares) and two commuting distances, the Maximum linear distance (m) travelled from cave entrance in KF 95%, and Maximum linear distance (m) travelled from the cave entrance to core foraging areas in KF 50%. These four parameters were initially explored for normality using the Shapiro-Wilk test, and the effects of sex, and body (forearm length in mm) and sample sizes on them were accessed by the Wilcoxon rank-sum test and Spearman correlations, respectively.

We implemented linear mixed effect models (LMMs) in a maximum likelihood framework [57] using these parameters as response variables and landscape treatments (conservation/disturbance), seasonality, and their interactions as fixed effects. Caves and species were modelled as nested random effects, and data for both species, *F*. *horrens* and *L*. *aurita*, in all caves were polled for these analyses. We used multi-model inferences to evaluate competing candidate models representing all possible combinations of these variables. Our model selection was based on the Akaike Information Criteria (AIC) considering all top-ranking models with ΔAIC < 2 as having similar support [58]. We adopted a stepwise modelling approach, firstly, selecting between random effects and using Restricted Maximum likelihood, then selecting between fixed effects using standard Maximum likelihood, and finally assessing estimates of the final models [57, 59].

All analyzes were conducted within the R environment [55], using “glmmTMB”, “ggplot2” and “MuMIn” packages [60–62]. Residual diagnostics of fitted models were accessed using the DHARMa package [63] and all models evaluated achieved convergence.

## Results

We successfully monitored sixteen individuals of *F*. *horrens* (length of forearm 34.9± 0.9 mm) and 14 of *L*. *aurita* (length of forearm: 48.8± 1.1 mm). Sampling lasted from three to four days on average for each individual of both species (*F*. *horrens*: 3.6 ± 1.86; *L*. *aurita*: 4.1 ± 1.17). We sampled a total of 1061 locations, and the mean number of locations obtained per individual was 42.7 ± 24.9 for *F*. *horrens* and 26.9 ± 13.6 for *L*. *aurita*. According to our initial exploratory analyses the parameters of space use were unrelated to the number of locations obtained and to the body size of bats for both species (Table 1 in S1 File). We also did not find relationships between the estimated parameters of space use and sex for both *F*. *horrens* or *L*. *aurita* (Table 1 in S1 File). Our exploratory results thus allowed us to pool individuals of both sexes of each of the two species for subsequent analyses, and results obtained for each individual sampled are summarized in Table 1.

### Habitat use/selection

**Fig 3:**
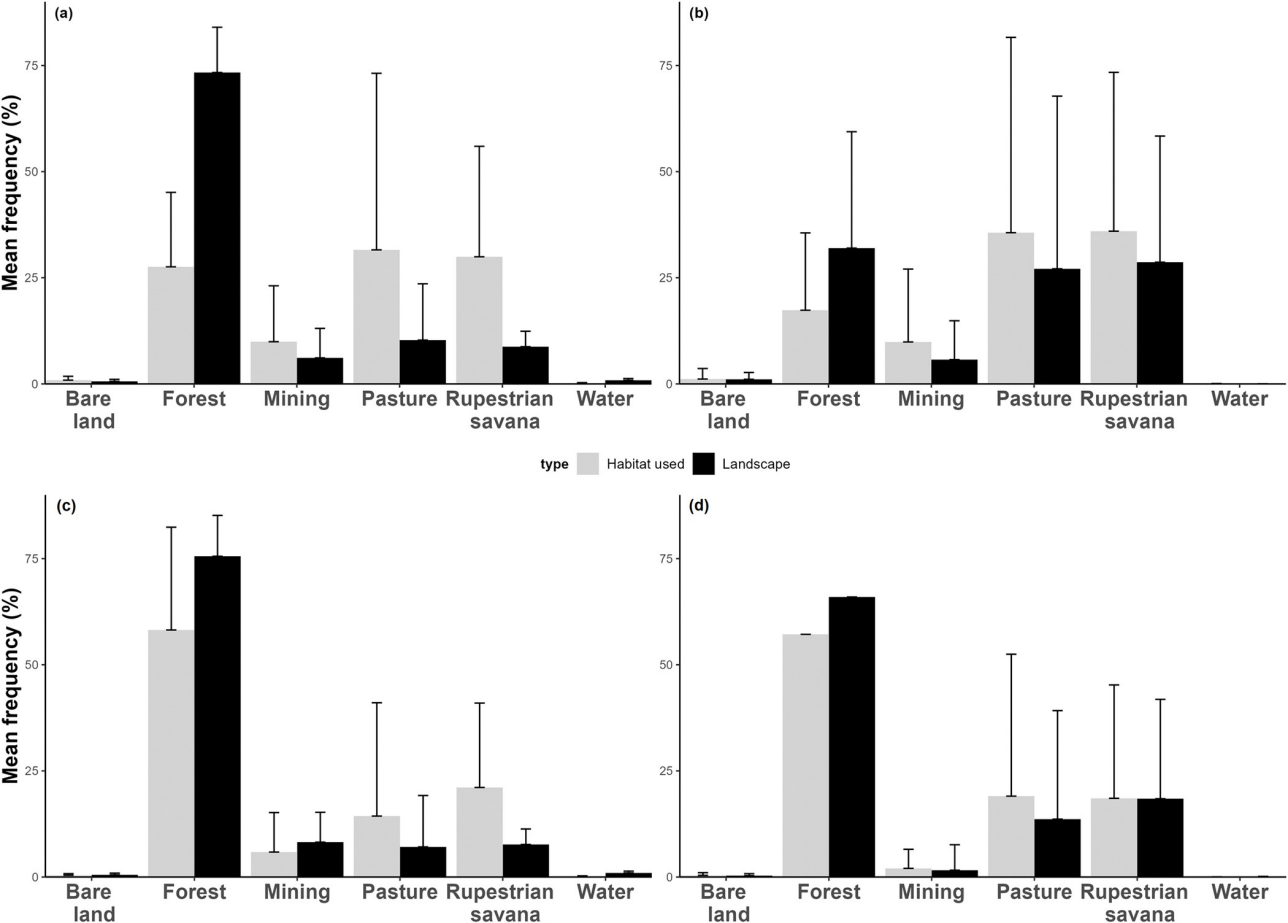
Landscape habitat composition (exposed bare land, forest, mining, pastures, rupestrian savannah, and water bodies) and habitat availability considering the estimate of KF 95% foraging area (a) and KF 50% foraging area (b) for *Furipterus horrens*, and KF 95% foraging area and KF 50% foraging area by *Lonchorhina aurita* (c) and (d).

Our habitat selection estimates for *F*. *horrens* indicate frequent foraging activities for this species in open spaces. When contrasting the KF 95% range area with the habitat availability (second-order selection), we observed that individuals of *F*. *horrens* preferentially used non-forested habitats, and mainly the canga and pastures, compared to their proportional use of any other type of habitat. There was also a pattern of avoidance of forest and water covered habitats (p = 0.0004, Fig 3A). The use of open areas appears only subtly when contrasting the foraging core area range KF50% and the MCP habitat availability (third-order selection) (p>0.05, Fig 3B).

On the other hand, the results for *L*. *aurita* individuals show that this species used all habitats proportionally to their availability in the landscape, except for their avoidance of water covered habitats (Second order selection, p = 0.005, Fig 3C). Furthermore, when contrasting the core foraging areas (KF50% defined) and the MCP habitat availability, we observed that *L*. *aurita* used all habitats proportionally to their availability in the landscapes (Third-order selection, p>0.05, Fig 3D). Forested habitats were the most used by this species as the rupestrian savannahs (cangas) and pastures appeared only as a complementary habitat use (Fig 3D).

### Use of the space

**Fig 4:**
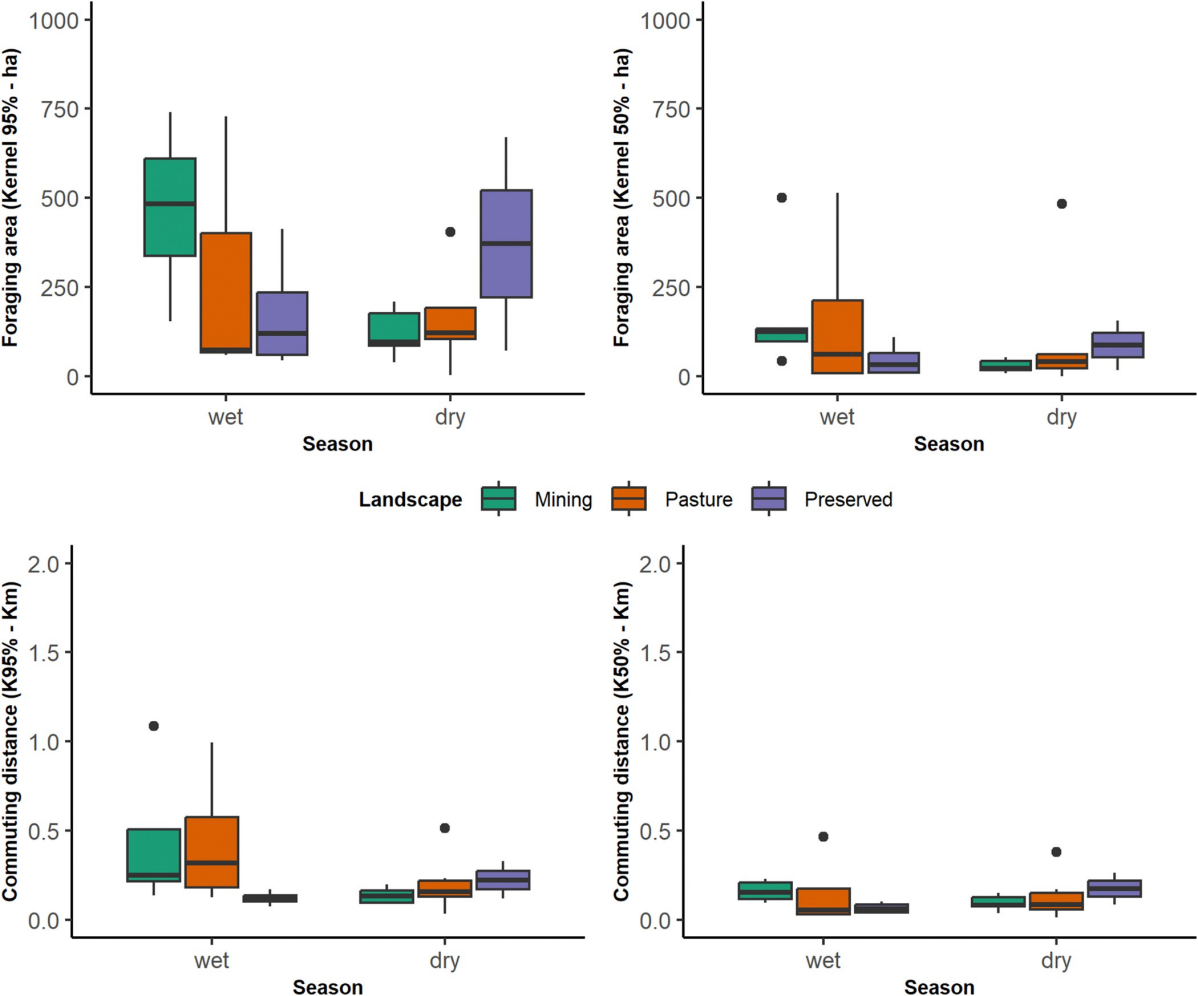
Effects of landscape composition and seasonality for the combined estimates of the foraging areas of *F*. *horrens* and *L*. *aurita* considering fixed kernel estimates, KF 95% (a) and the KF 50% (b), and the Maximum commuting distances in KF95% (c) and in KF50% (d).

The nested variable “Cave:Species” was selected as a random factor in the fixed slope model schemes for the adjusted models of all four response variables. Similarly, “Season” featured as a fixed effect between the most parsimonious models in all selection schemes (ΔAIC < 2), and the same happened with the Null model, hampering further interpretations. The exception was the Maximum commuting distance in KF95%, in which case the most parsimonious models included “Season” and the interaction between “Season” and “Landscape” (Table 2 in S1 File). Data on the use of disturbed landscapes (mining and pastures) indicate the accommodation of larger foraging areas and longer commuting distances compared to data from preserved landscapes in the wet season, and the inverse pattern is observable in the dry season (Fig 4). For the range area estimate models, there was no fixed effects narrowing the estimated foraging areas, either when KF 95% was considered or for KF 50% (Table 2 in S1 File).

On the other hand, the commuting distance models considering a range of KF 95% were affected by seasonality (β_dry_ = -0.9±0.32; p = 0.003), by “Landscape” (β_Preserved_ = -1.04±0.50; p = 0.04) and by the interaction between both (β_preserved:dry_ = 1.45±0.71; p = 0.04) highlighting different commuting distances in preserved landscapes, and between seasons (Table 3 in S1 File). Additionally, no fixed effects were found for commuting distance when considering a range of KF 50% (Table 2 in S1 File). Overall, our data indicate the influence of seasonality and of the landscape composition in the commuting distances flown for by these species, a pattern not observed for the bats’ foraging areas.

## Discussion

We found support for the prediction that landscape disturbances affect the commuting distances of *F*. *horrens* and *L*. *aurita*, and that this is mediated by seasonality. On the other hand, the estimated range areas for these two species were similar across preserved and anthropogenic landscapes. Moreover, we found support for our prediction of the differential use of habitats by the two bat species studied. We identified the selection of open areas for *F*. *horrens* and those included natural, preserved canga habitats, and the open degraded habitats represented by pastures. For *L*. *aurita*, forests were by far the most used habitats, and canga and pastures were only complementary as foraging areas.

A key point to our analysis of habitat preferences was the assumption that certain habitats may either be avoided or preferred relatively to others [56] and relative to what is available in the landscape. The sword-nosed bat *L*. *aurita* used proportionally most of what was available of forests, whereas the abundant forested habitats available for *F*. *horrens* were somewhat neglected by this species. Noteworthy results found for both species were that their use of space is markedly related to seasonality rather than to the landscape variation alone.

Data on the movement behavior of Neotropical bats in South America are scarce and include mostly estimates for phyllostomid species, such as nectar-feeding bat species monitored in the Brazilian Cerrado (*Glossophaga* and *Lonchophylla*) [21], fruit bats studied in the Brazilian Atlantic Forest (*Carollia*, *Artibeus*, and *Sturnira*) [20, 22], frugivores, aerial and foliage gleaning insectivores, and fish-eating bats radiotracked in the Tapajós region, Eastern Amazon´s (*Artibeus*, *Carollia*, *Gardnerycteris*, *Tonatia*, *Trachops*, and *Noctilio*) [23]. To our knowledge, we herein presented the first estimates of movement behavior for both *L*. *aurita* (Phyllostomidae), and for furipterids (Furipteridae), and that is why we cannot treat them in a broad comparative fashion.

Variations in habitat quality and availability, fine-tuned with specific foraging strategies, are expected to affect range area estimates as discussed elsewhere [19, 20, 64, 65]. Our estimates of area ranges and commuting distances for both species studied are larger, on average, than previous estimates found for other species of Neotropical bats [19–21, 23]. This is contrary to our expectations, particularly in the case of *F*. *horrens*, which is one of the tiniest Neotropical bats (3 to 4 g) as range sizes are expected to increase with body size and be limited by the energetic constraints in mammals [66, 67] including bats [68].

Our results suggest that these bats commute to forage differently in the pristine and disturbed landscapes of Carajás. They fly longer linear distances on disturbed landscapes in the wet season and shorter distances on preserved landscapes in the dry season (Fig 4). The influence of the seasonality, which modulates the bats’ responses to the landscape structures in Amazonia agrees with the general patterns previously described [24, 25, 69] primarily concerning to the spatiotemporal variability of food resources and the relative synchronicity of these fluctuations with the bats foraging activity and feeding behaviors. Seasonal reproductive and temporary environmental energetic demands require further investigation [26, 70]. Nonetheless, the two bat species studied appear to adjust the use of space to the observed changes in the landscape by accessing food resources similarly to their seasonal behavior. This may be evidenced by the range areas that remained similar between landscapes, by the larger effect size of “Season” on our models (Tables 1 and 2 in S1 File), and by the complementary use of anthropogenic habitats (Fig 3). Studies that evaluate the effects of mining on tropical bat communities are overall concurrent that natural habitat availability is a primary concern [71, 72], whereas studies on the movement of bats report their ability to adjust movements to explore fragmented landscapes [20, 21, 23]. We cannot make inferences about behavioral, ecological and physiological adjustments associated to the bats’ use of space based on the current data available, and further studies are necessary also to address these questions [1, 73].

All Neotropical bats use echolocation calls to some extent to navigate and search for food. The echolocation call patterns of *F*. *horrens* were recently described in more detail revealing that these bats emit short and extremely high frequency and broadband pulses that range from 70 to 210 kHz [74]. Based on the characteristics of the calls of *F*. *horrens*, resembling the calls of species of Old-World vespertilionid bats from the genus *Kerivoula* [75] Falcão and collaborators [74] suggested that they may also forage in dense vegetation. Our results appear to conflict with this suggestion because the combination of the movement patterns we found with the echolocation behavior described, is suggestive of patterns related to the *Edge Space Aerial foragers* guild as described by Denzinger et al. [76]. In terms of dietary preferences, the biology of *Furipterus horrens* is scarcely known [29, 74], and the little dietary data available recovered some evidence to the consumption of moths [29, 77]. Several species of moths have evolved sensitive ears to hearing the bats in one of the clearer examples of evolutionary predator-prey models [78]. Although along this “arms-race” some moths have evolved the capacity of hearing pulses much above 200 kHz, such as the case of greater wax moth, *Galleria mellonella* (Insecta: Lepidoptera) which can hear ultrasonic frequencies near 300 kHz [79] the combination of high frequency, low intensity and short calls of *F*. *horrens* may enable these bats to overcome the hearing abilities of moths [74], explaining, in part, these conflicting results.

*Lonchorhina aurita*, a foliage-gleaning, animalivorous phylostomid has been traditionally considered a narrow space forager and a “forest bat” [80]. As most animalivorous phyllostomids, bats from the genus *Lonchorhina* have been classified as passive gleaning foragers that would rely primarily on signs provided by their preys [76, 81]. However, several studies revealed refinements to our understanding of the evolution of foraging strategies and echolocation patterns of animalivorous phylostomids (e.g. *Macrophyllum macrophyllum*), including adaptations enabling then to increment their abilities to detect and locate prey with elaborated variations of echolocation calls, and some of them comparable to aerial insectivores from other bat families, such as Emballonuridae and Vespertilionidae [64, 82]. A recent study revealed that this is also the case for *L*. *aurita*, which has an unusual call structure among phyllostomids that deviates from the standard call design of bats of this family, and that may perhaps be related to its unique noseleaf, ear and tragus morphology. The echolocation system of *L*. *aurita* retains basic phyllostomid characteristics such as multi-harmonic FM-calls. On the other hand, as is typical in aerial insectivores, it has evolved to also include narrow-bandwidth CF-components and a terminal group [33]. Specialized foraging patterns among phylostomids have been more and more associated to the modulation of access to target resources and habitats [33].

Thus, by combining our results on habitat selection with the echolocation evidence available we can corroborate the suggestion of Gessinger et al. [33] of the evolution of an unusual foraging strategy for *L*. *aurita* consisting in a somewhat edge space aerial insectivore notably using primary forested habitats, and open habitats complementarily, accordingly to their availability. Our observations includes patterns of combined use of preserved and disturbed habitats, i.e. movements over fragmented landscapes that coincide with results obtained in other studies of insectivorous bats [64] and nectar feeding bats from the Cerrado [21] and poses further questions related to food constraints for both species, *F*. *horrens* and *L*. *aurita*. In central Amazonia, for instance, the foraging activity of the cave bat *Pteronotus rubiginosus* was mediated by insect availability, independent of the habitat types [83]. In a few words, a relevant point raised considering our results is the need to investigate how does the current mosaic of preserved and degraded habitats limit the food supply for these species. This is a key question because movements are costly, food sources are limiting, and all that information is important for the conservation and management programs of these bat species.

Food and roosts are key and limiting resources for bats [84] and ultimately, constraints to cave bats will affect cave ecosystems that largely depend upon the bat guano. This leads to the Brazilian policy concept “area of influence” of the cave, which would be an estimated space away from the cave perimeter, measured in kilometers needed to safeguard the biodiversity within the cave. Our incipient understanding of the mechanisms affecting the chances of Neotropical cave bats to effectively find and collect food in the surrounding landscapes, and the little knowledge about patterns of roosting selection of Neotropical cave bats prevents us from contributing with “area of influence” estimates, and that should be further investigated as it is crucial for bat and cave ecosystem conservation, and for exercises of prioritization for conservation.

## Supporting information

S1 File Results supporting analytical procedures. Includes Table1-Exploratory analyses of movement data, Table2-Model selection for Linear Mixed-effects Models (LMMs), Table3-Selected LMM estimates for Maximum commuting distance (K95%).
(XLSX)
